# Positive Phospho-Focal Adhesion Kinase in Gastric Cancer Associates With Poor Prognosis After Curative Resection

**DOI:** 10.3389/fonc.2022.953938

**Published:** 2022-08-02

**Authors:** Ke Peng, Suyao Li, Qian Li, Chenlu Zhang, Yitao Yuan, Menglin Liu, Lei Zhang, Yichen Wang, Shan Yu, Haisheng Zhang, Tianshu Liu

**Affiliations:** ^1^ Department of Medical Oncology, Zhongshan Hospital, Fudan University, Shanghai, China; ^2^ Center of Evidence-Based Medicine, Fudan University, Shanghai, China; ^3^ Department of Pathology, Zhongshan Hospital, Fudan University, Shanghai, China; ^4^ Department of Pathology, Fudan University Shanghai Cancer Center, Shanghai, China; ^5^ Department of General Surgery, Nanfang Hospital, Southern Medical University, Guangzhou, China

**Keywords:** phospho-FAK, gastric cancer, survival analysis, prognosis, nomogram

## Abstract

Gastric cancer (GC) is the fifth most commonly diagnosed cancer and usually has a dismal prognosis. Our previous study highlights the contribution of focal adhesion kinase (FAK) in the tumorigenesis of diffuse gastric cancer (DGC), a subtype of GC according to Lauren classification. The prognostic value of phosphorylated FAK (pFAK) in GC remains to be explored. To explore the prognostic value of pFAK, we retrospectively collected 176 formalin-fixed paraffin-embedded (FFPE) tumor tissues from GC patients who underwent D2 gastrectomy without neoadjuvant treatment. The immunohistochemistry (IHC) staining of pFAK was performed. Survival analysis was performed by Kaplan–Meier and risk factors were evaluated by Cox regression analysis. A pFAK-based nomogram was also constructed for the prediction of overall survival (OS). We demonstrated that the prognosis of pFAK-positive patients was worse than that of the pFAK-negative patients in GC (*p* = 0.010; hazard ratio [HR] = 1.777, 95% CI 1.131 to 2.791; median OS, 46.6 vs. 86.3 months, respectively), and positive pFAK was also an independent risk factor for the worse prognosis of GC (*p* = 0.0054; HR = 1.89, 95% CI 1.21–2.96). Moreover, the nomogram based on pFAK and other independent risk factors could improve predictive accuracy for prognosis of GC. In conclusion, through analysis of a large collection of clinically annotated GC samples, we demonstrate that pFAK is a negative prognostic factor in GC, and a nomogram integrating pFAK could help predict OS for GC patients.

## Introduction

Gastric cancer (GC) is the fifth most commonly diagnosed cancer and the fourth leading cause of cancer-related death worldwide according to GLOBOCAN 2020 (1). GC patients usually have a dismal prognosis when diagnosed. Therefore, it is important to explore the prognostic factor to distinguish the GC patients with high risk. According to Lauren classification, GC was mainly divided into two main histologic types, intestinal gastric cancer (IGC) and diffuse gastric cancer (DGC) (2). DGC is characterized by a highly invasive growth pattern where the tumor cells are poorly differentiated and lack cellular adhesion, hence leading to rapid invasion and metastases (3). In our previous study, we found that E-cadherin loss and *RHOA* Y42C mutation work together to activate FAK, which transforms normal murine gastric organoids to the DGC model of *Cdh1* ^-/-^ *RHOA* ^Y42C/+^ organoids. Our research nominated FAK as a potential therapeutic target for DGC (4).

FAK has complicated functions in cancers, which is both a non-receptor tyrosine kinase and a kinase-independent scaffold (5, 6). For the canonical activation of FAK as a non-receptor tyrosine kinase, FAK is recruited by the signaling from cell adhesion to form dimers, leading to the autophosphorylation of the tyrosine 397 (Y397) site. Then, FAK and SRC form the complex to fully activate FAK. Autophosphorylation of FAK on the Y397 site is the key step for the activation (7, 8).

FAK has been reported as a negative prognostic factor in several tumors. Higher expression of FAK indicates a worse prognosis in gliomas (9), hepatocellular carcinoma (HCC) (10), and non-small-cell lung cancer (NSCLC) (11). pFAK (Y397) was the active type of FAK, which can be detected by IHC staining. pFAK is also a risk factor in high-grade endometrial carcinoma (12) and glioma (9). In GC, it is reported that the recurrence-free survival (RFS) of GC patients with positive pFAK is also worse than that of the patients with negative pFAK. However, the sample size is relatively small in this research, just including 59 samples (13). Thus, it is necessary to explore the prognostic value of pFAK in GC with a larger sample size.

In our research, we retrospectively collected 176 FFPE GC samples and the relevant clinicopathological data in Zhongshan Hospital Fudan University and performed pFAK (Y397) staining. Then, the GC patients were divided into pFAK-positive and pFAK-negative groups. The survival analysis and Cox regression analysis were conducted to analyze the prognostic effect of pFAK. Additionally, we established the prognostic nomograms integrating pFAK and other independent risk factors to achieve more accurate predictions for the prognosis of GC patients.

## Materials and methods

### Patients and Collection of Clinical Data

A total of 176 GC patients who received D2 gastrectomy and adjuvant chemotherapy from March 2010 to May 2017 in Zhongshan Hospital Fudan University were recruited in this study. The inclusion criteria were as follows: (1) histologically proven gastric adenocarcinoma after radical gastrectomy with D2 lymph node dissection, (2) no neoadjuvant chemotherapy or radiotherapy, (3) no evidence of metastatic disease, (4) pathological stage II to III GC according to the 8th edition of the AJCC cancer staging manual, (5) IGC or DGC according to Lauren classification, and (6) no synchronous or metachronous cancer. The patients with positive resection margin, M1 lymph node, and distant metastases were excluded from our study. The clinicopathological data were collected including sex, age, nerve invasion, lymphovascular invasion (LVI), tumor deposit, Lauren classification, and TNM stage. The TNM stage was evaluated according to the 8th edition of the AJCC cancer staging manual. Overall survival (OS) was recorded as the time from surgery to the death of the patient or the last follow-up time (October 2021).

This study was performed with the approval of the Ethics Committee of Zhongshan Hospital Fudan University (B2020-171). All patients were enrolled with informed consents.

### Immunohistochemistry (IHC)

IHC staining for the slide GC samples was conducted using an automated system (BenchMark XT, Roche). pFAK (Y397 site) antibody (44-624G, Invitrogen) at a dilution of 1:50 was used. pFAK staining was evaluated by an experienced gastrointestinal pathologist without access to clinical data (LZ). The staining intensity was evaluated by H-score. The intensity of staining is classified as 0 to 3 (0: negative; 1: weak; 2: intermediate; 3: strong). In each case, H-score with a potential range of 0–3 was calculated as follows: H-score= (1 × percentage of weakly stained cells) + (2 × percentage of moderately stained cells) + (3 × percentage of strongly stained cells). Samples with H-score < 0.25 were assigned to the pFAK-negative group, and those H-score ≥ 0.25 were allocated to the pFAK-positive group.

### Statistical Analysis

The correlations between pFAK and clinicopathologic variables were analyzed using Pearson’s *χ* ^2^ test or Fisher’s exact test as appropriate. Kaplan–Meier curve and log-rank test were used for survival analysis. Cox proportional hazards regression model was used to evaluate the risk factors. Survival analysis and Cox analysis were performed by R packages of “tableone”, “survival”, and “survminer”. The nomogram based on independent risk factors for the prediction of OS was established. Calibration curve, decision curve analysis (DCA), receiver operating characteristic curve (ROC curve), and concordance index (C-index) were further used to evaluate its predictive performance as previously described (14–16). Nomogram and calibration curve were constructed by the R package “rms”. ROC curve and C-index were constructed by the R package “survival”. *p*-value less than 0.05 was considered as a statistically significant difference.

## Results

### Correlations Between pFAK and Clinicopathologic Characteristics in GC

**Figure 1:**
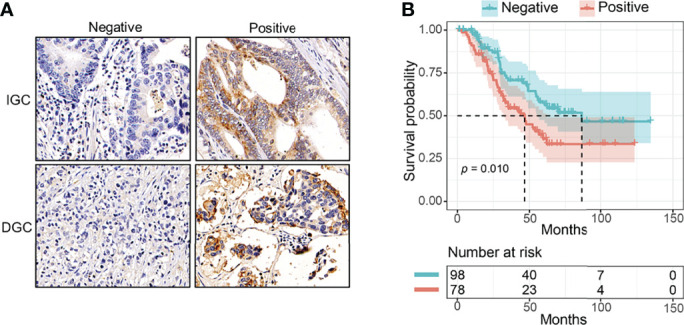
The prognostic value of pFAK in GC patients. **(A)** Representative immunohistochemistry of positive or negative pFAK in GC paraffin samples. **(B)** Kaplan–Meier curves for pFAK-positive or -negative group in GC (*p* = 0.010; HR = 1.777, 95% CI 1.131 to 2.791; median OS, 46.6 vs. 86.3 months, respectively). *p*-values were calculated by log-rank test. The number of patients at risk are below the survival curve.

To explore the prognostic value of pFAK in GC, we evaluated pFAK (Tyr397 site), marking active FAK, by IHC staining in a surgically resected GC cohort of 176 patients with curative intent. Representative pFAK-negative or pFAK-positive staining results are shown in **Figure 1A**. Seventy-eight patients were assigned to the pFAK-positive group, and 98 patients were allocated to the pFAK-negative group. The associations between clinicopathologic characteristics and pFAK were analyzed. We found that positive pFAK was statistically associated with age > 60 (*p* = 0.015), but not other clinicopathologic characteristics (**Table 1**).

**Table 1:** Correlation between pFAK and clinicopathologic characteristics in GCs.

Characteristics	Subgroup	No. of Cases (%)	pFAK		*p*-value
			Negative (*n* = 98)	Positive (*n* = 78)	
Sex	Female	39 (22.2)	21	18	0.937
	Male	137 (77.8)	77	60	
Age	>60	78 (44.3)	35	43	0.015
	≤60	98 (55.7)	63	35	
Nerve invasion	Negative	58 (33.0)	36	22	0.301
	Positive	118 (67.0)	62	56	
LVI	Negative	71 (40.3)	45	26	0.125
	Positive	105 (59.7)	53	52	
Tumor deposit	Negative	110 (62.5)	63	47	0.695
	Positive	66 (37.5)	35	31	
Lauren	Intestinal	87 (49.4)	49	38	0.695
	Diffuse	89 (50.6)	49	40	
T stage	T1	1 (0.6)	0	1	0.196
	T2	10 (5.7)	7	3	
	T3	80 (45.5)	39	41	
	T4	85 (48.3)	52	33	
N stage	N0	18 (10.2)	8	10	0.746
	N1	27 (15.3)	15	12	
	N2	40 (22.7)	24	16	
	N3	91 (51.7)	51	40	
TNM stage	II	39 (22.2)	19	20	0.418
	III	137 (77.8)	79	58	

GC, gastric cancer; LVI, Lymphovascular invasion; p-values were calculated using Pearson’s χ^2^ test or Fisher’s exact test.

### The Prognostic Role of pFAK Expression in GC Patients

**Figure 2:**
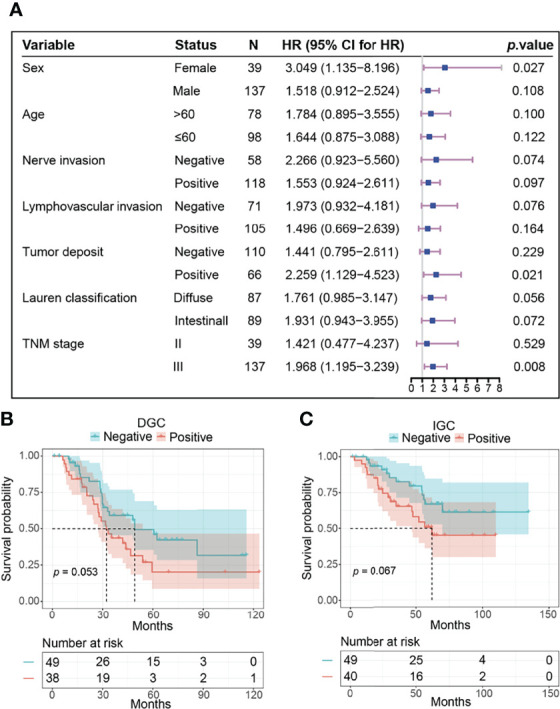
The prognostic value of pFAK in subtypes of GC. **(A)** Results of survival analysis in different subgroups. HR and p value was calculated by univariate Cox regression. The reference level in each subgroup was negative pFAK. **(B, C)** Kaplan-Meier curves of pFAK-positive and pFAK-negative GCs in DGC subgroup **(B)** and IGC subgroup **(C)**. Log-rank test was used to calculate p value for Kaplan-Meier curves. The number of patients at risk are below the survival curve.

Then, we compared the OS of pFAK-positive and pFAK-negative groups by Kaplan–Meier curve. The survival analysis showed that the OS of the pFAK-positive group was significantly shorter than that of the pFAK-negative group in GC (*p* = 0.010; hazard ratio [HR] = 1.777, 95% CI 1.131 to 2.791; median OS, 46.6 vs. 86.3 months, respectively) (**Figure 1B**). Moreover, the prognostic value of pFAK in subgroups was also analyzed by univariate Cox regression (**Figure 2A**). We found that pFAK was also the negative prognostic factor in the subgroups of positive tumor deposit (*p* = 0.021; HR = 2.259, 95% CI 1.129 to 4.523) and stage III GC patients (*p* = 0.008; HR = 1.968, 95% CI 1.195 to 3.239). The OS of the pFAK-positive group was worse than that of the pFAK-negative group in the subgroups of positive tumor deposit (*p* = 0.018; median OS, 27.4 vs. 55.9 months, respectively) and stage III GC patients (*p* = 0.007; median OS, 34.6 vs. 68.8 months, respectively) (**Figures S1A, C**), but not the subgroups of negative tumor deposit and stage II GC patients (**Figures S1B, D**). The OS of the pFAK-positive group was also worse than that of the pFAK-negative group in subgroups of DGC and IGC, though not statistically significant (DGC, *p* = 0.056; IGC, *p* = 0.072) (**Figures 2B, C**). These data demonstrated a robust prognostic value of pFAK level in GC patients.

### Univariate and Multivariate Cox Analyses of pFAK and other Risk Factors

To further assess the prognostic value of pFAK for GC patients, univariate and multivariate analyses in this cohort were performed. Positive nerve invasion, lymphovascular invasion and tumor deposit, DGC, stage III, and positive pFAK were significantly associated with worse OS of GC patients after gastrectomy in the univariable Cox proportional hazards model (**Table 2**). Furthermore, multivariable Cox model analysis showed that positive pFAK (*p* = 0.040; HR = 1.66, 95% CI 1.02 to 2.69), as well as positive tumor deposit (*p* = 0.042; HR = 1.66, 95% CI 1.02 to 2.72) and DGC (*p* = 0.019; HR = 1.79, 95% CI 1.10 to 2.94) were all confirmed as the independent risk factors for predicting OS (**Table 2**).

**Table 2:** Univariate and multivariate Cox analysis of clinicopathological factors associated with the survival in GCs.

Variants	Number of cases (%)	Univariate analysis		Multivariate analysis	
		HR (95% CI)	*p*-value	HR (95% CI)	*p*-value
Sex					
Female	39 (22.2)	Ref		Ref	
Male	137 (77.8)	1.04 (0.61–1.76)	0.896	0.89 (0.52–1.53)	0.666
Age					
>60	78 (44.3)	Ref		Ref	
≤60	98 (55.7)	0.73 (0.47–1.15)	0.172	0.75 (0.47–1.21)	0.246
Nerve invasion					
Negative	58 (33.0)	Ref		Ref	
Positive	118 (67.0)	1.74 (1.05–2.90)	**0.033**	1.39 (0.82–2.38)	0.225
LVI					
Negative	71 (40.3)	Ref		Ref	
Positive	105 (59.7)	1.81 (1.14–2.88)	**0.012**	1.29 (0.78–2.12)	0.319
Tumor deposit					
Negative	110 (62.5)	Ref		Ref	
Positive	66 (37.5)	1.81 (1.15–2.83)	**0.010**	1.66 (1.02–2.72)	**0.042**
Lauren					
Intestinal	89 (50.6)	Ref		Ref	
Diffuse	87 (49.4)	1.98 (1.26–3.13)	**0.003**	1.79 (1.10–2.94)	**0.019**
TNM stage					
II	39 (22.2)	Ref		Ref	
III	137 (77.8)	2.02 (1.11–3.67)	**0.021**	1.40 (0.72–2.73)	0.317
pFAK					
Negative	98 (55.7)	Ref		Ref	
Positive	78 (44.3)	1.79 (1.14–2.80)	**0.011**	1.66 (1.02–2.69)	**0.040**

GC, gastric cancer; HR, hazard ratio; CI, confidential interval; LVI, lymphovascular invasion. Data were obtained from Cox proportional hazards model.Bold means the p value less than 0.05, which was statistically significant.

### Nomograms for Predicting OS of GC Patients after Gastrectomy

**Figure 3:**
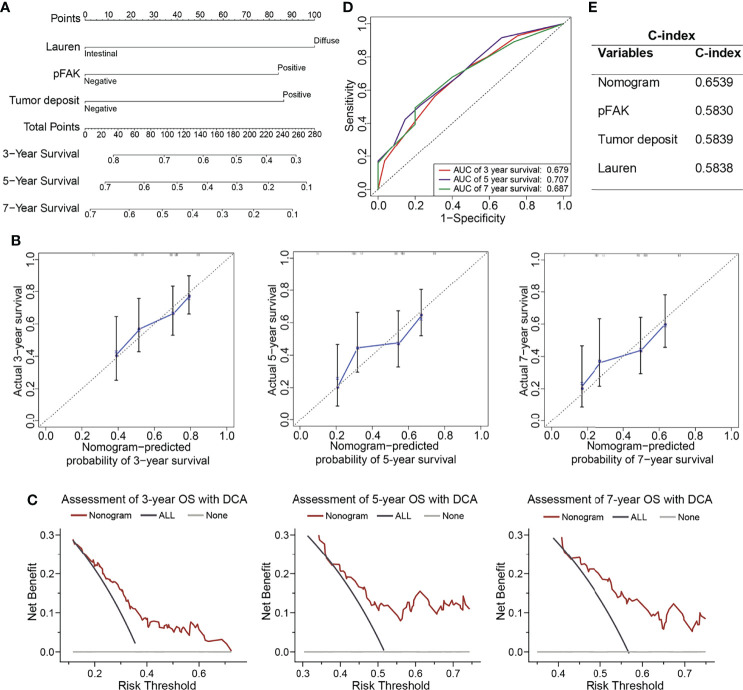
Construction of prognostic nomograms in GCs. **(A)** The nomogram was constructed to predict overall survival (OS) for GC patients. The value of each variable (Lauren classification, pFAK and tumor deposit) was added to get the total points. The probability of 3-, 5-, or 7-year OS could be calculated by drawing a vertical line from the total point axis to the probability scale. **(B)** Calibration curves for 3-, 5-, and 7-year OS of GC patients. The actual OS and the nomogram-predicted probability of OS are plotted on the *y*-axis and *x*-axis, respectively. The dotted line along 45° represents a perfect consistency between observed and predictive values. **(C)** Calibration curves for 3-, 5-, and 7-year OS of GC patients. **(D)** ROC curves for 3-, 5-, and 7-year OS of GC patients. **(E)** C-index for nomogram, Lauren classification, pFAK or tumor deposit.

To obtain a better prognostic model for clinical practice, we constructed a nomogram for the prediction of survival at 3, 5, and 7 years after gastrectomy by integrating pFAK, tumor deposit, and DGC, which were the independent prognostic factors according to multivariate Cox analysis (**Figure 3A**). Then, we performed calibration curves to test the accuracy of the nomogram. It was shown that the predictive survival of pFAK-based nomograms at 3, 5, and 7 years matched the actual observation well (**Figure 3B**). The DCA curves showed good prediction effect of the nomogram (**Figure 3C**). The AUCs of 3-year, 5-year, and 7-year survival were 0.679, 0.707, and 0.687, respectively, in ROC curves (**Figure 3D**). To compare the predictive accuracy of the nomogram with the individual variants, the C-index of the nomogram, pFAK, tumor deposit, and Lauren classification was calculated. We found that the nomogram model had better predictive performance of survival than pFAK, tumor deposit, and Lauren classification alone with the C-index of 0.6539 in the nomogram (**Figure 3E**).

## Discussion

FAK pathway has multiple functions in gastric cancer. In our previous study, we found that Cdh1 (E-Cadherin) loss and RHOA Y42C mutation work together to activate FAK and then transform the normal gastric epithelial cells to DGC. This study revealed FAK as a potential therapeutic target for DGC (4). FAK pathway promotes the migration and invasion of GC cancer cells (17, 18). CXCL1 promotes lymph node metastasis of GCs by activating integrin β1-FAK-AKT signaling (18). Furthermore, activation of the FAK-YAP pathway induces cisplatin resistance in gastric cancer (19). FAK silencing also enhances the therapeutic efficacy of 5-FU (20).

The canonical activation of FAK begins from the signaling from cell adhesions (17). When activated by integrins, inactive FAK forms dimers, leading to the autophosphorylation of tyrosine 397 (Y397) site (7). Then, FAK forms the complex with SRC to be fully activated (7, 8). Activated FAK regulates multiple biological cellular functions, including cell survival, migration, and invasion of cancer cells (8).

The phosphorylation of FAK (Tyr397 site) is a surrogate marker to evaluate the activation of FAK (8). pFAK has been established to be a prognostic factor in several cancers, including endometrial carcinoma (12) and glioma (9). In GC, prior reports found no significant difference between the recurrence-free survival (RFS) based on assessment of total FAK; however, the RFS of patients in the positive pFAK group was found to be shorter than the patients whose tumors lack pFAK (13). However, the sample size of this prior study was relatively small, and the prognostic value of pFAK in subgroups, such as IGC or DGC, was not analyzed. In our larger cohort, we found that positive pFAK was associated with worse prognosis in stage II/III surgically resected GC not treated neoadjuvantly according to survival analysis. Multivariant Cox analysis also showed that positive pFAK was the independent risk factor in GC. These data also supported our previous finding that pFAK was a potential therapeutic target in GC patients (4).

Our previous research has shown the key function of FAK pathway in the tumorigenesis of DGC. In the subgroup of DGC, the prognosis of pFAK-positive patients was also worse than that of the pFAK-negative patients, which also indicates that FAK activation promotes the malignancy of DGC. Moreover, the pFAK-positive patients also had worse prognosis in the subgroup of IGC, though not statistically significant. These data demonstrate that FAK activation is also an important regulator in IGC, which needs further research.

Recently, the nomogram has been widely used to construct effective prognostic models for tumors. In our research, we used pFAK and other independent risk factors of GC to establish a nomogram for the prediction of survival. Our nomogram showed better performance than pFAK, tumor deposit, and Lauren classification alone for the likelihood of 3-year, 5-year, and 7-year OS for GC patients according to C-index. Therefore, our pFAK-based nomogram was a convenient and accurate tool to predict the prognosis of GC patients after gastrectomy.

Of course, our research had limitations. Firstly, our study just included stage II and stage III GC patients after radical gastrectomy. Whether pFAK is the prognostic factor in metastatic GC still needs to be explored. Secondly, our study is a retrospective research conducted only in a single institution, making it necessary to perform multicenter, prospective studies to further validate our results. Lastly, pFAK is the phosphorylated protein, which might degrade after long-term preservation.

## Conclusions

In conclusion, our research found that positive pFAK was associated with worse OS, and it was also an independent risk factor for GC patients after curative resection. Our study further constructed a nomogram by integrating pFAK and other independent risk factors, which improved predictive accuracy for GC prognosis.

## Data Availability Statement

The original contributions presented in the study are included in the article/**Supplementary Material**, further inquiries can be directed to the corresponding authors.

## Ethics Statement

The studies involving human participants were reviewed and approved by ethics committee of Zhongshan Hospital Fudan University. The ethics committee waived the requirement of written informed consent for participation.

## Author Contributions

Conception and design: TL, HZ, and KP. Acquisition of data: KP, SL, and QL. Analysis and interpretation of data: KP, YY, ML, LZ, and YW. Writing, review, and/or revision of the manuscript: TL, KP, and SL. Study supervision: TL and HZ. All authors contributed to the article and approved the submitted version.

## Funding

This study was granted by the National Natural Science Foundation of China (82172925) and the Shanghai Sailing Program (19YF1407100).

## Acknowledgments

We would like to thank Dr. Ning Pu for the valuable suggestions about statistical analysis.

## Conflict of Interest

HZ is the founder of Signet Therapeutics.

The remaining authors declare that the research was conducted in the absence of any commercial or financial relationships that could be construed as a potential conflict of interest.

## Publisher’s Note

All claims expressed in this article are solely those of the authors and do not necessarily represent those of their affiliated organizations, or those of the publisher, the editors and the reviewers. Any product that may be evaluated in this article, or claim that may be made by its manufacturer, is not guaranteed or endorsed by the publisher.

## References

[1] Sung H Ferlay J Siegel RL Laversanne M Soerjomataram I Jemal A. Global Cancer Statistics 2020: GLOBOCAN Estimates of Incidence and Mortality Worldwide for 36 Cancers in 185 Countries. CA: Cancer J Clin (2021) 71(3):209–49. doi: 10.3322/caac.21660 33538338

[2] Lauren P. The Two Histological Main Types of Gastric Carcinoma: Diffuse and So-Called Intestinal-Type Carcinoma. An Attempt at a Histo-Clinical Classification. Acta Pathologica Microbiologica Scandinavica (1965) 64:31–49. doi: 10.1111/apm.1965.64.1.31 14320675

[3] Kakiuchi M Nishizawa T Ueda H Gotoh K Tanaka A Hayashi A. Recurrent Gain-of-Function Mutations of RHOA in Diffuse-Type Gastric Carcinoma. Nat Genet (2014) 46(6):583–7. doi: 10.1038/ng.2984 24816255

[4] Zhang H Schaefer A Wang Y Hodge RG Blake DR Diehl JN. Gain-Of-Function RHOA Mutations Promote Focal Adhesion Kinase Activation and Dependency in Diffuse Gastric Cancer. Cancer Discov (2020) 10(2):288–305. doi: 10.1158/2159-8290.cd-19-0811 31771969 PMC7007383

[5] Kleinschmidt EG Schlaepfer DD. Focal Adhesion Kinase Signaling in Unexpected Places. Curr Opin Cell Biol (2017) 45:24–30. doi: 10.1016/j.ceb.2017.01.003 28213315 PMC5482783

[6] Naser R Aldehaiman A Diaz-Galicia E Arold ST. Endogenous Control Mechanisms of FAK and PYK2 and Their Relevance to Cancer Development. Cancers (2018) 10(6):196. doi: 10.3390/cancers10060196 PMC6025627 29891810

[7] Acebrón I Righetto RD Schoenherr C de Buhr S Redondo P Culley J. Structural Basis of Focal Adhesion Kinase Activation on Lipid Membranes. EMBO J (2020) 39(19):e104743. doi: 10.15252/embj.2020104743 32779739 PMC7527928

[8] Sulzmaier FJ Jean C Schlaepfer DD. FAK in Cancer: Mechanistic Findings and Clinical Applications. Nat Rev Cancer (2014) 14(9):598–610. doi: 10.1038/nrc3792 25098269 PMC4365862

[9] Ding L Sun X You Y Liu N Fu Z. Expression of Focal Adhesion Kinase and Phosphorylated Focal Adhesion Kinase in Human Gliomas is Associated With Unfavorable Overall Survival. Trans Res (2010) 156(1):45–52. doi: 10.1016/j.trsl.2010.05.001 20621036

[10] Itoh S Maeda T Shimada M Aishima S Shirabe K Tanaka S. Role of Expression of Focal Adhesion Kinase in Progression of Hepatocellular Carcinoma. Clin Cancer Res (2004) 10(8):2812–7. doi: 10.1158/1078-0432.ccr-1046-03 15102689

[11] Ji HF Pang D Fu SB Jin Y Yao L Qi JP. Overexpression of Focal Adhesion Kinase Correlates With Increased Lymph Node Metastasis and Poor Prognosis in Non-Small-Cell Lung Cancer. J Cancer Res Clin Oncol (2013) 139(3):429–35. doi: 10.1007/s00432-012-1342-8 PMC11824597 23143646

[12] Zhou J Roh JW Bandyopadhyay S Chen Z Munkarah AR Hussein Y. Overexpression of Enhancer of Zeste Homolog 2 (EZH2) and Focal Adhesion Kinase (FAK) in High Grade Endometrial Carcinoma. Gynecologic Oncol (2013) 128(2):344–8. doi: 10.1016/j.ygyno.2012.07.128 22871469

[13] Lai IR Chu PY Lin HS Liou JY Jan YJ Lee JC. Phosphorylation of Focal Adhesion Kinase at Tyr397 in Gastric Carcinomas and Its Clinical Significance. Am J Pathol (2010) 177(4):1629–37. doi: 10.2353/ajpath.2010.100172 PMC2947260 20724588

[14] Pu N Yin H Zhao G Nuerxiati A Wang D Xu X. Independent Effect of Postoperative Neutrophil-to-Lymphocyte Ratio on the Survival of Pancreatic Ductal Adenocarcinoma With Open Distal Pancreatosplenectomy and Its Nomogram-Based Prediction. J Cancer (2019) 10(24):5935–43. doi: 10.7150/jca.35856 PMC6856566 31762803

[15] Peng K Chen E Li W Cheng X Yu Y Cui Y. A 16-mRNA Signature Optimizes Recurrence-Free Survival Prediction of Stages II and III Gastric Cancer. J Cell Physiol (2020) 235(7-8):5777–86. doi: 10.1002/jcp.29511 32048287

[16] Zhou C Chen W Sun J Atyah M Yin Y Zhang W. Low Expression of WW Domain-Containing Oxidoreductase Associates With Hepatocellular Carcinoma Aggressiveness and Recurrence After Curative Resection. Cancer Med (2018) 7(7):3031–43. doi: 10.1002/cam4.1591 PMC6051234 29905011

[17] Bae IH Yoon SH Lee SB Park JK Ho JN Um HD. Signaling Components Involved in Bcl-W-Induced Migration of Gastric Cancer Cells. Cancer Lett (2009) 277(1):22–8. doi: 10.1016/j.canlet.2008.11.022 19097687

[18] Wang Z Wang Z Li G Wu H Sun K Chen J. CXCL1 From Tumor-Associated Lymphatic Endothelial Cells Drives Gastric Cancer Cell Into Lymphatic System *via* Activating Integrin β1/FAK/AKT Signaling. Cancer Lett (2017) 385:28–38. doi: 10.1016/j.canlet.2016.10.043 27832972

[19] Uchihara T Miyake K Yonemura A Komohara Y Itoyama R Koiwa M. Extracellular Vesicles From Cancer-Associated Fibroblasts Containing Annexin A6 Induces FAK-YAP Activation by Stabilizing β1 Integrin, Enhancing Drug Resistance. Cancer Res (2020) 80(16):3222–35. doi: 10.1158/0008-5472.Can-19-3803 32605995

[20] Hou J Tan Y Su C Wang T Gao Z Song D. Inhibition of Protein FAK Enhances 5-FU Chemosensitivity to Gastric Carcinoma *via* P53 Signaling Pathways. Comput Struct Biotechnol J (2020) 18:125–36. doi: 10.1016/j.csbj.2019.12.010 PMC6961071 31969973

