# Supplementary figures and images for: Positive Phospho-Focal Adhesion Kinase in Gastric Cancer Associates With Poor Prognosis After Curative Resection
Source: Front Oncol. 2022 Aug 2;12:953938. doi: 10.3389/fonc.2022.953938 (PMC9379279; doi:10.3389/fonc.2022.953938)

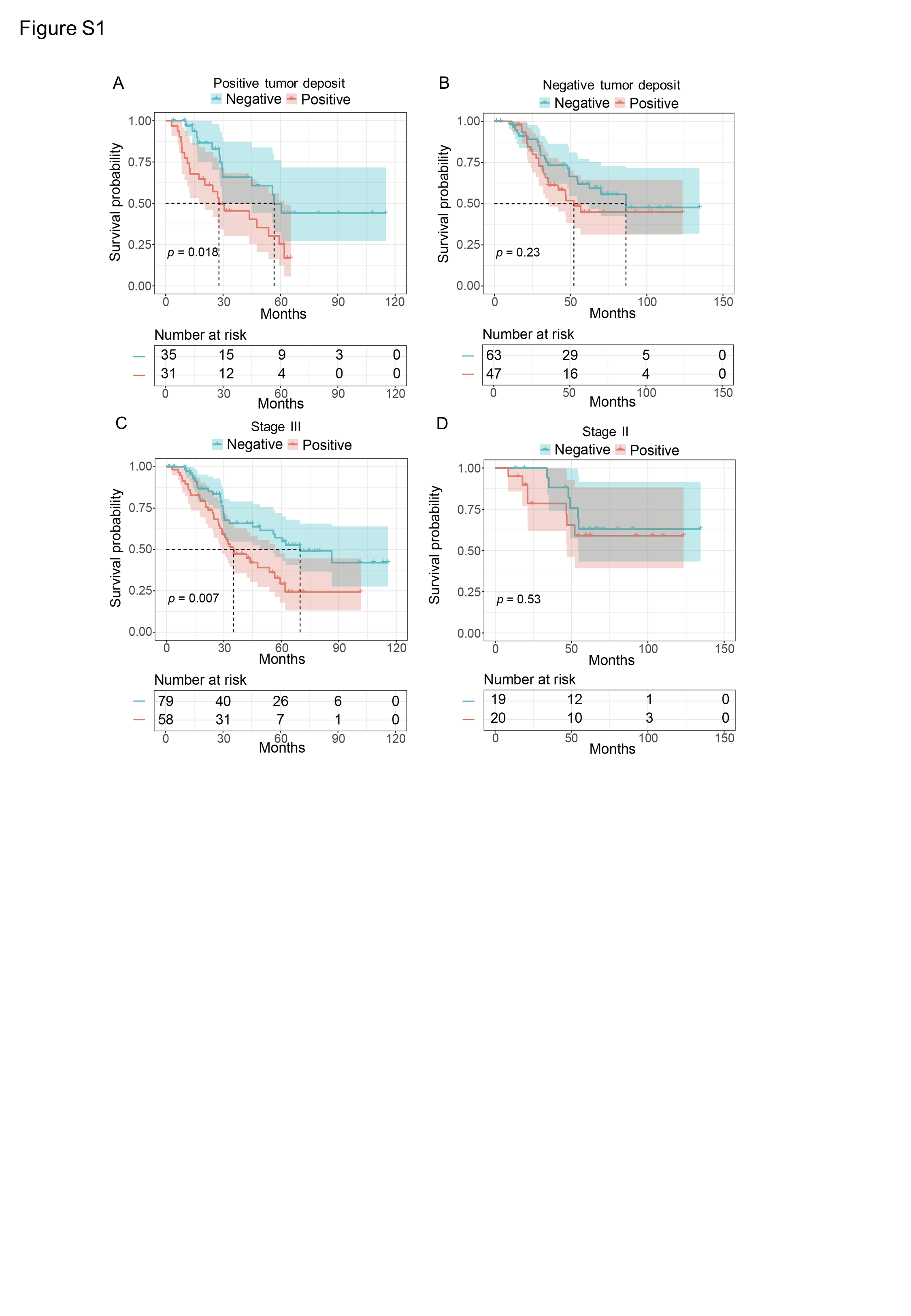

Supplement: Supplementary file 1 [file Image_1.tif]
